# Pulsed‐Laser and Mechanical Reduction of Graphene Oxide Combined with NiCoFeMoW High‐Entropy Alloys for Electrocatalytic Oxygen Evolution Reaction

**DOI:** 10.1002/cssc.202500466

**Published:** 2025-06-29

**Authors:** Hossein Mahdavi, Omer Şamil Akcan, Yağız Morova, M. Barış Yağcı, Uğur Ünal, Hadi Jahangiri

**Affiliations:** ^1^ Materials Science and Engineering Koç University Sariyer Istanbul 34450 Turkey; ^2^ Department of Physics Istanbul Technical University Maslak Istanbul 34469 Turkey; ^3^ Koç University Surface Science and Technology Center (KUYTAM) Koç University Sariyer 34450 Istanbul Turkey; ^4^ Department of Chemistry Koç University Sariyer 34450 Istanbul Turkey; ^5^ Koç University Hydrogen Technologies Center (KUHyTech) Koç University Sariyer 34450 Istanbul Turkey

**Keywords:** high‐entropy alloys, high‐energy ball milling, oxygen evolution reaction, pulsed‐ laser irradiation, reduced graphene oxide

## Abstract

The development of cost‐effective and high‐performance electrocatalysts for the oxygen evolution reaction is critical for sustainable energy conversion technologies. In this study, graphene oxide is subjected to two distinct reduction techniques: nanosecond pulsed‐laser irradiation and high‐energy ball‐milling. Structural characterization reveals that laser treatment led to partial reduction, while mechanical treatment achieves a higher degree of reduction. The treatments induce morphological transformations, with laser‐irradiated samples exhibiting localized “wrinkling” due to thermal effects, whereas high‐energy ball‐milling induced “folding” resulted from prolonged mechanical stress. The electrocatalytic performance of reduced graphene oxide is further enhanced by incorporating a NiCoFeMoW high‐entropy alloy, prepared by mechanical alloying technique. Electrochemical evaluation demonstrated that the heterostructures exhibited superior electrocatalytic activity, achieving an overpotential of 141.8 mV at 10 mA·cm^−^^2^ for the best sample. These findings underscore the potential of reduced graphene oxide‐supported high‐entropy alloys as a promising alternative to noble‐metal‐based electrocatalysts, offering a scalable and environment‐friendly approach for advancing water‐splitting technologies.

## Graphical abstract

High‐entropy alloy (HEA)‐reduced graphene oxide (rGO) heterostructures are synthesized via pulsed laser and mechanical reduction of graphene oxide. Incorporation of NiCoFeMoW HEAs significantly enhances oxygen evolution reaction activity, with overpotentials as low as 141.8 mV. This study presents a scalable, cost‐effective, and environmentally friendly strategy for designing high‐performance, noble‐metal‐free electrocatalysts for sustainable water‐splitting technologies.

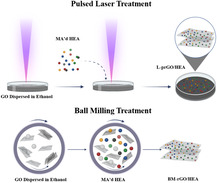

## 1 Introduction

Hydrogen production through water splitting is a promising renewable energy technology for addressing the global energy crisis and environmental challenges.^[^ ^1^, ^2^ ^]^ As a clean, sustainable energy carrier with zero greenhouse gas emissions and high energy density, hydrogen offers significant potential for mitigating climate change.^[^ ^3^ ^]^ However, the efficiency of water splitting is hindered by the slow kinetics of the oxygen evolution reaction (OER), a complex four‐electron process occurring at the anode.^[^ ^4^, ^5^, ^6^ ^]^ Unlike the hydrogen evolution reaction (HER) at the cathode, OER requires higher overpotentials, posing a significant challenge to developing efficient, large‐scale hydrogen production technologies.^[^ ^1^ ^]^ Noble metal‐based catalysts, such as iridium (Ir) and ruthenium (Ru) oxides, are currently the most effective OER electrocatalysts.^[^ ^1^, ^4^ ^]^ However, their high costs, limited abundance, and poor long‐term stability restrict their practical applications, prompting a shift toward non‐noble metal alternatives.^[^ ^7^ ^]^

High‐entropy alloys (HEAs) have emerged as a promising class of alternatives for noble‐metal OER electrocatalysts due to their unique properties.^[^ ^8^, ^9^ ^]^ Composed of five or more metals in molar ratios ranging from 5% to 35%,^[^ ^10^ ^]^ HEAs exhibit high configurational entropy and sluggish diffusion kinetics, which enhance their resistance to over‐oxidation and improve the structural stability.^[^ ^11^, ^12^, ^13^ ^]^ Additionally, lattice distortion and “cocktail effect” enable tunable electronic structures and enhance their catalytic activity.^[^ ^14^ ^]^ Moreover, HEAs possess complex compositions and structures that create unique atomic distributions and binding sites. These features enable tunable binding energies through elemental ratio adjustment, offering more active sites for adsorbing reactants and intermediates involved in HER and OER compared to traditional materials.^[^ ^15^ ^]^ Recent studies have demonstrated significant reductions in OER overpotentials with HEAs, such as FeCoNiMo, achieving values as low as 250 mV–89 mV lower than conventional IrO_2_ catalysts.^[^ ^16^ ^]^ In another study, Li et al.^[^ ^17^ ^]^ prepared amorphous FeCoNiCuYP/C high‐entropy phosphide/carbon structures by employing a high‐entropy metal organic framework as a template with multiple catalytically active sites. The composites reveal superior OER performance with high stability at an overpotential of 316 mV to reach a current density of 100 mA·cm^−2^, due to the fine‐tuned electronic structure because of the corporation of Fe, Ni, and Co and their synergistic interactions. Our prior work further shows that Mo and Mn doping in CoCuFeNi‐based HEAs achieved OER overpotentials of 375 ± 15 mV, comparable to advanced non‐noble metal catalysts.^[^ ^18^ ^]^ In our latest work, pulsed laser deposited NiCoFeCuMoMnO_x_ high‐entropy oxide electrocatalysts were employed as OER electrocatalysts, exhibiting η_10_ values as low as 180 mV, showing the potential of laser treatment on the OER performance of HEAs.^[^ ^19^ ^]^

To further enhance the OER performance of HEAs, integrating them with conductive, high‐surface‐area nanomaterials such as reduced graphene oxide (rGO) is a promising strategy.^[^ ^20^, ^21^, ^22^, ^23^, ^24^ ^]^ rGO offers high conductivity, large specific surface area, and effective anchoring capabilities, making it ideal for supporting electrocatalysts.^[^ ^25^ ^]^ Unlike graphene oxide (GO), which suffers from poor electrical conductivity due to oxygen‐containing groups, rGO restores the sp^2^ graphitic lattice and enhances conductivity through reduction.^[^ ^25^, ^26^ ^]^ Among various reduction methods such as chemical,^[^ ^27^ ^]^ electrochemical,^[^ ^28^ ^]^ thermal^[^ ^29^ ^]^ approaches, laser ablation and mechanically milling stand out for moderate operational temperatures, and environmental friendliness.^[^ ^30^, ^31^ ^]^

In this study, we have developed novel rGO/HEA heterostructures for OER applications using nanosecond pulsed laser and high‐energy ball milling (HEBM). GO was treated by laser irradiation, followed by the introduction of HEAs (NiCoFeMoW) and additional laser irradiation. This innovative two‐step laser ablation process ensured strong interaction between HEA nanoparticles and the rGO matrix, promoting uniform dispersion, reducing particle size, and increasing specific surface area. In parallel, HEBM was employed on GO to obtain BM‐rGO. Later on, HEA powders were introduced to the BM‐rGO mixture, and further ball milled to obtain BM‐rGO/HEA heterostructures.

The results highlight that **rGO/HEA** materials achieved OER overpotentials as low as 141.9 mV (BM‐rGO/HEA) and 160.6 mV (L‐prGO/HEA), significantly outperforming many existing electrocatalysts. This scalable and efficient fabrication strategy underscores the potential of rGO‐supported HEAs as cost‐effective, high‐performance alternatives to noble‐metal‐based electrocatalysts for sustainable water splitting.

## 2 Experimental Section

### 2.1 Preparation of HEAs

The NiCoFeMoW HEA powders were prepared by mechanical alloying using RetschPM 200 high‐energy planetary ball‐mill equipment as described in our previous work.^[^ ^18^ ^]^ Briefly, equal weights of pure metallic powders (99.9%) were mixed and loaded into the mill under an inert argon atmosphere inside a glovebox to hinder oxidation during the dry stage of milling. Subsequently, the mixture was subjected to two separate 10 h of dry and wet (ethanol medium) milling at a 400‐rpm rate. Ball‐to‐powder ratio of 10:1 was chosen. The powders were collected and dried at room temperature.

### 2.2 GO Treatment

GO powder with a thickness of 2–5 layers, average diameter of 4.5 μm, and a specific surface area of 420 m^2^/g was employed as a support material (Nanografi, Turkey). Two different methods were utilized for the reduction of GO: (1) nanosecond pulse laser irradiation and (2) high‐energy ball‐milling. For the laser treatment, a Q‐switched Nd:YAG laser with an integrated single‐pass amplifier (Spectra‐Physics Quanta‐Ray) was used. This laser system, with a nanosecond pulse duration, operated at a power of 150 mJ and emitted light at a wavelength of 532 nm. To do so, 5 mg of GO was dispersed in ethanol and irradiated with the pulsed laser for a duration of 6 h. In the mechanical milling method, the same concentration of GO in ethanol was processed by HEBM for 6 h at a speed of 400 rpm using the same high‐energy planetary ball mill machine.

### 2.3 Preparation of GO/HEA Heterostructures

Following these two methods, HEA powders were added to the processed GO, and the laser irradiation and milling process was continued separately for an additional hour with the same parameters. It is noteworthy that the weight of the milling balls was measured before and after the process, confirming that the weight loss was negligible. However, trace amounts of some elements from the balls and the jar, including Fe could be introduced into the system, which was controlled by EDS mappings.

### 2.4 Structural Characterization

The microstructure of the samples was examined using a Carl Zeiss Ultra Plus field emission scanning electron microscope (FE‐SEM) equipped with both secondary and backscattered electron detectors. The crystal structure of processed GO was analyzed using a Bruker D2 Phaser X‐ray diffractometer (XRD) equipped with Cu Kα radiation (λ = 1.5405 Å) over a 2θ range of 5°–80°. Raman spectroscopy measurements were performed using a Renishaw Invia Raman microscope. X‐ray photoelectron spectroscopy (XPS) analysis was conducted with a Thermo Scientific K‐α spectrometer equipped with an Al Kα monochromator source (1486.6 eV), and the data was processed using Avantage 5.9 software, with all spectra calibrated to the C 1s peak at 284.5 eV. Fourier transform infrared (FTIR) spectra were recorded using a Thermo Scientific iS 10 FT‐IR spectrometer. The microstructure and crystallinity of the samples were examined using an aberration‐corrected high‐resolution transmission electron microscope (HRTEM, Hitachi HF5000), operating at 200 kV.

### 2.5 Electrochemical Measurements

The electrochemical characteristics of the samples were examined using linear sweep voltammetry (LSV) with a Biologic VSP Potentiostat in a conventional three‐electrode setup. In this system, a mercury/mercury oxide (Hg/HgO) electrode served as the reference and a graphite rod was used as the counter electrode, both in a 1 M KOH medium. As for working electrode, a glassy carbon (GC) electrode (3 mm diameter) was modified with the prepared electrocatalysts. Briefly, 870 μL of ethylene glycol was combined with 130 μL of a 1 wt% Nafion solution for preparing the catalyst ink. This mixture was then blended with 5 mg of catalyst powder and subjected to ultrasonic treatment for 60 min to ensure uniform distribution. Afterwards, 3 μL of the catalyst ink was deposited onto a pre‐cleaned GC electrode and was dried at 60 °C for 6 h in an air environment. LSV measurements were recorded in a potential range between 1.2 and 1.8 V (vs reversible hydrogen electrode, RHE) for the oxygen evolution reaction (OER), in an oxygen‐saturated electrolyte to maintain O_2_/H_2_O stability and equilibrium at 1.23 V versus RHE. The electrocatalytic activities were assessed at a current density of 10 mA·cm^−2^. Potentials measured against the Hg/HgO electrode were converted to RHE potentials using the formula E_RHE_ = E_Hg/HgO_  + 0.059 pH + 0.098. Stability tests were performed with a rotating disk electrode (RDE) at 1600 rpm using chronopotentiometry technique at current density of 10 mA·cm^−^^2^. Electrochemical impedance spectroscopy (EIS) measurements were conducted to further investigate the charge transfer properties of the electrodes. For these tests, a sinusoidal potential of 10 mV was applied over a frequency range of 100 kHz–0.1 Hz to obtain Nyquist plots. Additionally, cyclic voltammetry (CV) was used to determine the double‐layer capacitance (C_dl_) and estimate the electrochemically active surface area (ECSA). CV tests were conducted at scan rates of, 20, 40, 60, 80, and 100 mV·s^−1^ across a potential range of 1.1–1.2 V versus RHE.

## 3 Result and Discussion

**Figure 1:**
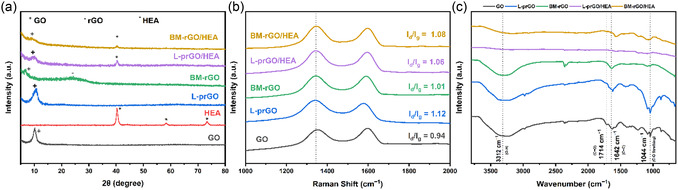
a) XRD patterns, b) Raman spectra, and c) FTIR spectra of GO and processed GO after laser exposure and milling, with and without HEA addition.

In this study, the reduction of GO was investigated using two distinct methods: pulsed‐laser irradiation and mechanical milling. The XRD patterns of GO‐based samples in ethanol, before and after these treatments, are shown in **Figure** **1**a. Initially, the XRD spectrum of GO displayed a prominent peak at 2θ = 10.5° ± 0.02, corresponding to an interlayer spacing of 1.20 ± 0.02 Å, attributed to the presence of oxygen‐containing functional groups such as hydroxyl, carboxyl, and epoxy groups, which intercalate between the carbon layers. After 6 h of mechanical milling, the characteristic peak at 2θ = 10.50° ± 0.02 disappeared, and a broad peak appeared at 2θ = 25.31° ± 0.02 with 3.22 ± 0.02 Å d‐ spacing. This change can be attributed to the milling process, which effectively removed some of the oxygenated functional groups.^[^ ^31^ ^]^ The elimination of these groups during mechanical milling indicates the production of rGO. In contrast, after 6 h of laser irradiation at a wavelength of 532 nm, the peak at 2θ = 10.50° ± 0.02 shifted slightly to 2θ = 10.70° ± 0.02, becoming broader and less intense. This shift likely indicates a partial reduction of GO, where some oxygenated functional groups were removed, leading to decreased peak intensity and broadening. The incomplete removal of these groups during laser irradiation suggests the formation of partially reduced GO (prGO).^[^ ^32^, ^33^, ^34^ ^]^

After incorporating NiCoFeMoW HEA powders into GO following a 6‐hour reduction process through milling and laser irradiation, an additional hour of the same treatment was applied. XRD analysis revealed a decrease in the intensity of the peak at 2θ = 10.75° ± 0.01 in prGO processed by laser exposure, and 2θ = 25.36° ± 0.02 in rGO produced by HEBM, likely due to the further removal of oxygen‐containing functional groups. Furthermore, distinct XRD peaks at 2θ = 41.21° ± 0.01, 58.70° ± 0.01, and 73.82° ± 0.01 with 2.17 ± 0.01, 1.57 ± 0.01, and 1.28 ± 0.01°A d‐spacing confirmed the presence of the HEA powders, corresponding to the (110), (200), and (211) planes of the body‐centered cubic (BCC) crystal structure, indicating successful integration of the powders into the rGO matrix while maintaining their crystalline integrity.

The structural transformations of GO to prGO or rGO through either pulsed‐laser irradiation or HEBM are evident in the Raman spectroscopy results. Figure 1b presents the Raman spectra for GO and GO subjected to pulsed‐laser irradiation, as well as GO treated by HEBM. In the Raman spectrum of GO, the G band appears at 1586 cm^−1^, which corresponds to the ordered sp^2^‐bonded carbon atoms, and the D band is located at 1336 cm^−1^, associated with edge planes and disordered carbon structures.^[^ ^35^, ^36^ ^]^ Similarly, the Raman spectrum of the pulsed‐laser irradiated GO exhibits G and D bands at 1584 cm^−1^ and 1327 cm^−1^, respectively.^[^ ^35^, ^36^ ^]^ The milled GO also shows the G and D bands at 1582 cm^−1^ and 1325 cm^−1^, respectively.^[^ ^31^ ^]^ It is noteworthy that the frequencies of the G and D bands in the pulsed‐laser irradiated GO are nearly identical to those in the as‐prepared GO.^[^ ^34^ ^]^ After adding HEA powders and conducting an additional hour of reduction, the G and D band values were 1582 cm^−1^ and 1326 cm^−1^ for laser radiation, and 1581 cm^−1^ and 1324 cm^−1^ for milling, respectively. This minimal shift reflects the short processing duration after powder addition. From the Raman spectra in Figure 1b, the D/G intensity ratio (I_d_/I_g_) before and after the reduction processes (laser irradiation and milling) are observed to be 0.94, 1.12, and 1.01, respectively. After adding the powder and applying an additional hour of processing, the values changed to 1.06 for L‐prGO/HEA and 1.08 for BM‐rGO/HEA, respectively. The I_d_/I_g_ ratio serves as an indicator of the degree of disorder and the average size of the sp^2^ domains.^[^ ^31^, ^34^, ^35^, ^36^ ^]^

FTIR spectra which is shown Figure 1c was employed to examine the changes in functional groups on prGO of laser‐irradiated GO and rGO produced by HEBM. The absorption peak at 1642 cm^−1^, attributed to carbon‐carbon (C = C) double bonds, is characteristic of pristine graphene. Distinct peaks for GO were observed at 1044 cm^−1^ for C—O stretching and 1714 cm^−1^ for C = O carbonyl stretching, alongside a prominent *O*–H stretching peak at 3312 cm^−1^. Following milling treatment, a decrease in the C—O peak and the disappearance of the C = O peak was noted, indicating a reduction of GO. In contrast, laser irradiation led to an increase in the C—O peak and the retention of the C = O peak. Additionally, a reduction in the *O*–H stretching peak at 3312 cm^−1^ suggests the formation of reduced graphene oxide.^[^ ^31^, ^34^, ^35^, ^36^ ^]^ Based on these FTIR spectra results, it can be inferred that GO undergoes reduction through different functional groups in both methods.

**Figure 2:**
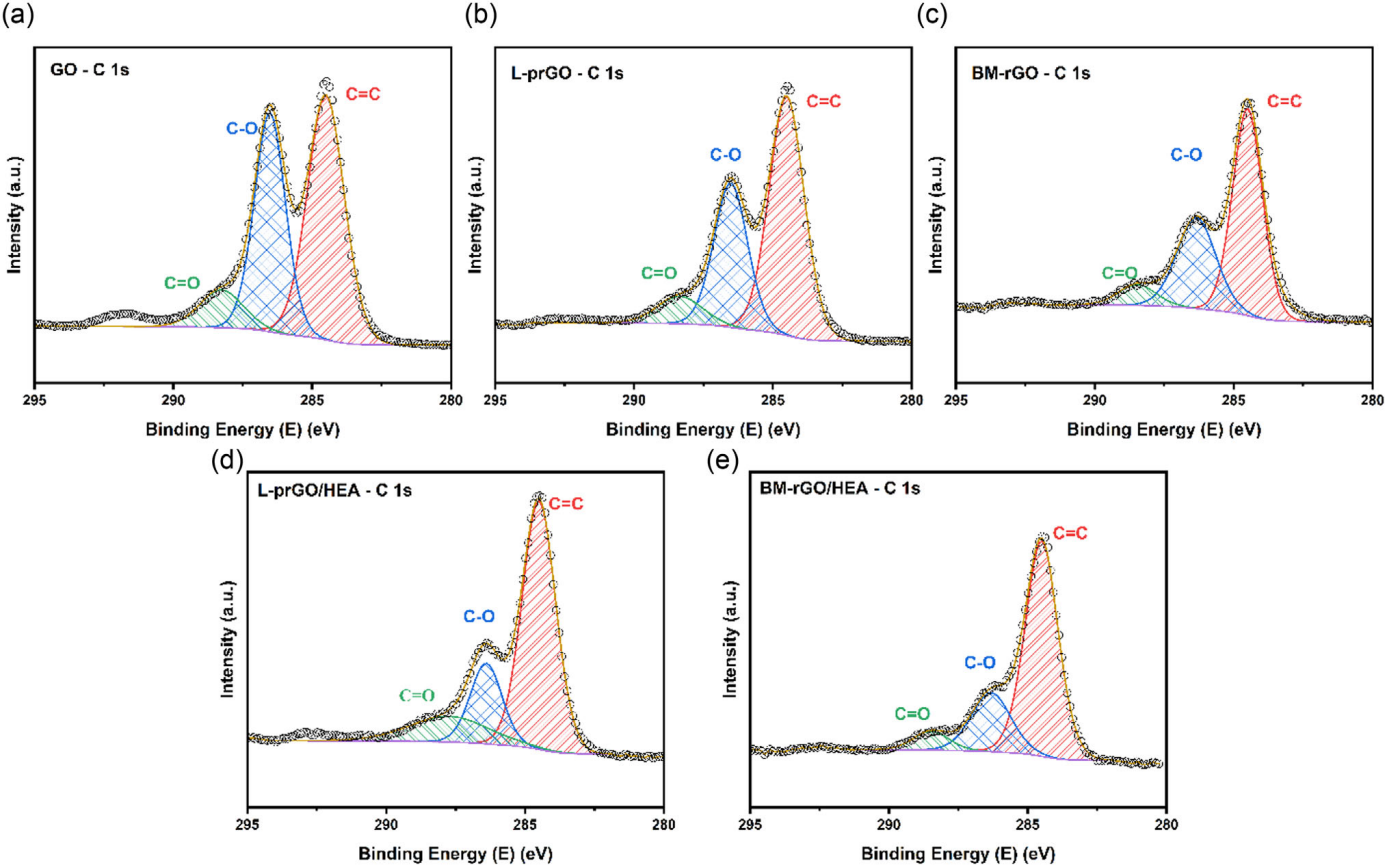
High‐resolution XPS C 1s spectra of a) GO, b) L‐prGO, c) BM‐ rGO, d) L‐prGO/HEA, and e) BM‐rGO/HEA.

**Figure** **2** presents the high‐resolution XPS spectra of the C1s region in GO before and after treatment by milling and laser irradiation. All spectra were calibrated to the C—C peak position at 284.5 eV. Due to the limited resolution of the XPS spectrometer, it was not possible to distinguish between the C—C and C = C peaks, so they are considered a single signal for comparison with peaks corresponding to carbon atoms bonded with other functional groups.^[^ ^37^ ^]^ The C 1s spectra can be accurately fitted with three peaks cantered at ≈284.5 eV (peak 1), 286 eV (peak 2), and 288 eV (peak 3), corresponding to C=C (sp^2^), C—O (epoxy and alkoxy), and C=O groups, respectively. In the untreated GO (Figure 2a), the peaks primarily consist of two main components associated with C=C and C—O, alongside a minor component related to C=O. The intensity of the C—O component is significantly reduced following treatment with milling and laser exposure, as shown in Figure 2b,c. As shown in Figure 2 d and Figure 2e, the addition of HEAs to the system does not affect the oxidation state significantly. As shown in **Table** **1**, XPS analysis of GO in the C 1s region reveals a significant degree of oxidation, indicating the presence of various oxygen‐containing functional groups, such as carbonyl, epoxy, and hydroxyl groups. The peaks corresponding to covalent bonds between carbon and oxygen atoms are more intense in GO compared to GO treated by milling or laser exposure. The CC/CO intensity ratio of GO (1.07) is lower than that of reduced GO obtained by laser treatment (1.20) or by milling (1.97), where “CC” refers to the combined contribution of C—C and C = C bonds, and “CO” represents all carbon–oxygen bond configurations. These findings are consistent with the XRD and Raman spectroscopy results, confirming that the GO was successfully reduced by both milling and laser exposure processes. However, the GO degree of reduction was higher in milling method than the laser irradiation technique.

**Table 1:** The C 1s peak positions of GO before and after treatment with laser and HEBM.

	GO		L‐prGO		BM‐rGO		L‐prGO/HEA		BM‐rGO/HEA	
	Peak Position	Peak Intensity	Peak Position	Peak Intensity	Peak Position	Peak Intensity	Peak Position	Peak Intensity	Peak Position	Peak Intensity
C1s Scan A(C‐C and C = C)	284.50	51.67	284.47	54.47	284.48	66.32	284.50	64.46	284.48	70.26
C1s Scan B (C—O)	286.52	38.86	286.51	35.23	286.41	23.41	284.65	19.81	286.41	22.96
C1s Scan C(C = O)	288.27	9.46	288.35	10.30	289.38	10.26	289.24	15.73	289.38	6.78

In order to investigate the surface chemistry of GO/HEA heterostructures and their effect on the electrocatalytic activity of the compounds, high‐resolution spectra of the active sites, that is, metallic elements and O 1s were obtained and presented in Figure S2, Supporting Information. Comprehensive XPS analysis conducted before and after the long‐term OER stability measurements reveals that the oxidation states of the metal sites in the BM‐rGO/HEA catalyst are mostly preserved, indicating that direct redox cycling of the metal centers does not play a dominant role in the catalytic process. Nonetheless, the electrode surface undergoes slight reconstruction, primarily reflected in the loss of lower oxidation states of Ni and W. More specifically, high‐resolution Ni 2p spectra displays four distinct peaks in the MA'd HEA sample, corresponding to metallic Ni, Ni^2+^, Ni^3+^, and the satellite peaks. Most notably, it is observed that after laser treatment and long‐term OER tests, the metallic Ni peak disappears, which points out the oxidative nature of these processes. Co 2p spectra, as shown in Figure S2b, Supporting Information, consists of Co^2+^and Co^3+^ peaks, without displaying much change after treatments and measurements. Fe 2p spectra displays the same satellite and main peaks after laser processing, ball‐milling, and OER measurements, which is consistent with the already oxidized state of Fe in the mechanically alloyed HEA samples. Mo 3 d spectra displays peaks corresponding to Mo^4+^ and Mo^6+^, without going through much change after treatment and long‐term electrochemical analysis. On the contrary, W 4f spectra of the Lp‐rGO and BM‐rGO/HEA after stability tests revealed that lower oxidation state of W^4+^ is converted to higher states such as W^5+^ and W^6+^. This suggests that during OER, the leaching of W is contributing to the surface reconstruction, exposing the highly active Ni, Co, and Fe sites.^[^ ^38^ ^]^ Another noticeable change appears in the O 1s spectra (Figure S2f, Supporting Information), where an increase in the M—O (metal–oxygen) peak is observed alongside a reduction in the M–OH (metal–hydroxide) signal following the OER process. This shift suggests the elimination of surface –OH groups and their conversion into more stable M–O bonds under oxidative conditions. Such a transformation points to a mild surface reconstruction, involving chemical and structural reorganization of the catalyst's outer atomic layers, which enhances coordination variability and facilitates improved electron transport.^[^ ^19^, ^39^ ^]^ This reorganization facilitates the generation or stabilization of catalytically active M–O sites, which are crucial for promoting the oxygen evolution reaction by improving both adsorption capabilities and electron transfer efficiency.^[^ ^40^ ^]^

**Figure 3:**
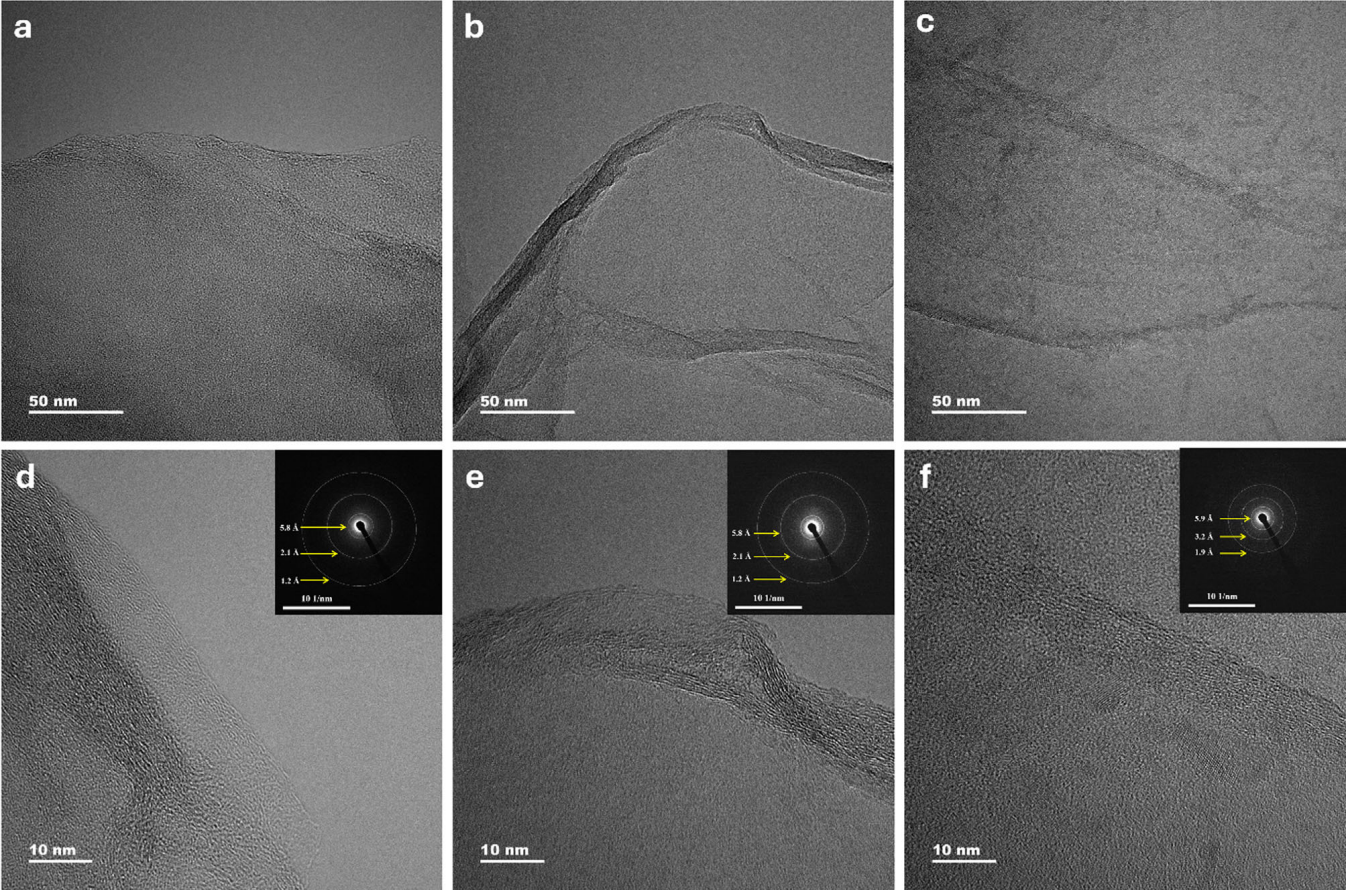
TEM images of a) GO, b) rGO by Laser, c) rGO by Milling. HR‐TEM and corresponding SAED patterns (insets) of d) GO, e) L‐prGO and f) BM‐rGO.

**Figure** **3** shows the HRTEM images and selected area electron diffraction (SAED) patterns of GO, L‐prGO, and BM‐rGO samples. The flake morphology of the GO can be seen in the Figure 3a. The wrinkled and folded structures in rGO nanosheets, produced either by laser exposure and milling, are clearly visible in Figure 3b,c. During the reduction process, the removal of oxygen‐containing functional groups leads to two distinct structural forms: wrinkled and folded configurations. “Wrinkling” refers to smaller, irregular surface deformations, which is due to rapid, localized thermal effects, while “folding” denotes larger, more organized bends or creases a result of extended mechanical stress over time, leading to more significant structural rearrangement.^[^ ^41^, ^42^ ^]^ The wrinkled structure is more prominent in L‐prGO, while folding is more pronounced in BM‐rGO. These structural features arise from the removal of oxygen groups, which induces relaxation and contraction within the GO matrix, leading to the formation of wrinkles and folds. The presence of these features serves as confirmation of the effective removal of oxygen functional groups during the reduction process. The electron diffraction rings obtained from the SAED patterns of GO (Figure 3d), L‐prGO (Figure 3e), and BM‐rGO (Figure 3f) reveal interplanar d‐spacings characteristic of graphene. The GO sample displays interplanar distances of 2.10 ± 0.02 Å and 1.20 ± 0.01 Å, closely matching the values reported by Saxena et al.^[^ ^43^ ^]^ and Ceniceros et al.^[^ ^35^ ^]^ Similarly, L‐prGO exhibits d‐spacings of 2.09 ± 0.01 Å and 1.18 ± 0.01 Å, while BM‐rGO shows a slightly reduced interplanar distance of 1.92 ± 0.02 Å. This reduction in d‐spacing values compared to GO indicates a partial restoration of the graphene lattice during the reduction process, aligning with the XRD results. Moreover, a ring corresponding to a d value of 3.2 Å, indicative of the reduced phase in the milling method, was observed, as also reported by Calderón‐Ayala et al.^[^ ^31^ ^]^ This observation is consistent with the formation of multilayer graphene, where the interlayer distance approaches the graphite‐like value of 0.34 nm, as noted by Pendolino et al.^[^ ^44^ ^]^ The removal of oxygen‐containing functional groups during the reduction of GO facilitates the recovery of graphitic domains. Concurrently, folding of the GO sheets may occur, forming crystalline regions with short‐range order or semi‐parallel arrangements in the folded areas. These regions often manifest as discrete diffraction spots in the HRTEM‐SAED pattern. The effectiveness of laser exposure and milling in the reduction process is further evidenced by the formation of graphitic regions within the reduced samples. Calderón‐Ayala et al. highlighted that techniques like high‐energy ball milling not only promote reduction but also allow the generation of laminar structures with nanometric thickness, a feature confirmed by the present study.^[^ ^31^ ^]^ The FE‐SEM images of GO, HEA, and the heterostructures were obtained and displayed in Figure S3, Supporting Information. Figure S3a, Supporting Information, shows the stacked GO sheets before treatment and Figure S3b, Supporting Information, displays the plate‐like structure of NiCoFeMoW‐based HEA sample with a lateral size of ≈2.5 μm. As shown in Figure S3c,d, Supporting Information, after laser irradiation and ball milling with the presence of HEA, GO sheets are covered by the HEA particles. Additionally, the treatments give rise to a porous structure with a high surface area and more available active sites. The cavities and pores found on the BM‐rGO/HEA sample are smaller in size and display finer features, resulting in a higher specific surface area, as shown later in ECSA calculations, which suggests them as a favorable candidate for electrocatalysis. As depicted in Figure S3e, Supporting Information, the morphology of the BM‐rGO/HEA sample appears to be of a more porous nature. This variation could possibly be attributed to the further oxidation and leaching of HEA elements, specifically W, during long‐term tests, which illustrates the possible mild surface reconstruction during OER.^[^ ^38^ ^]^

## 4 Electrochemical Measurements

**Figure 4:**
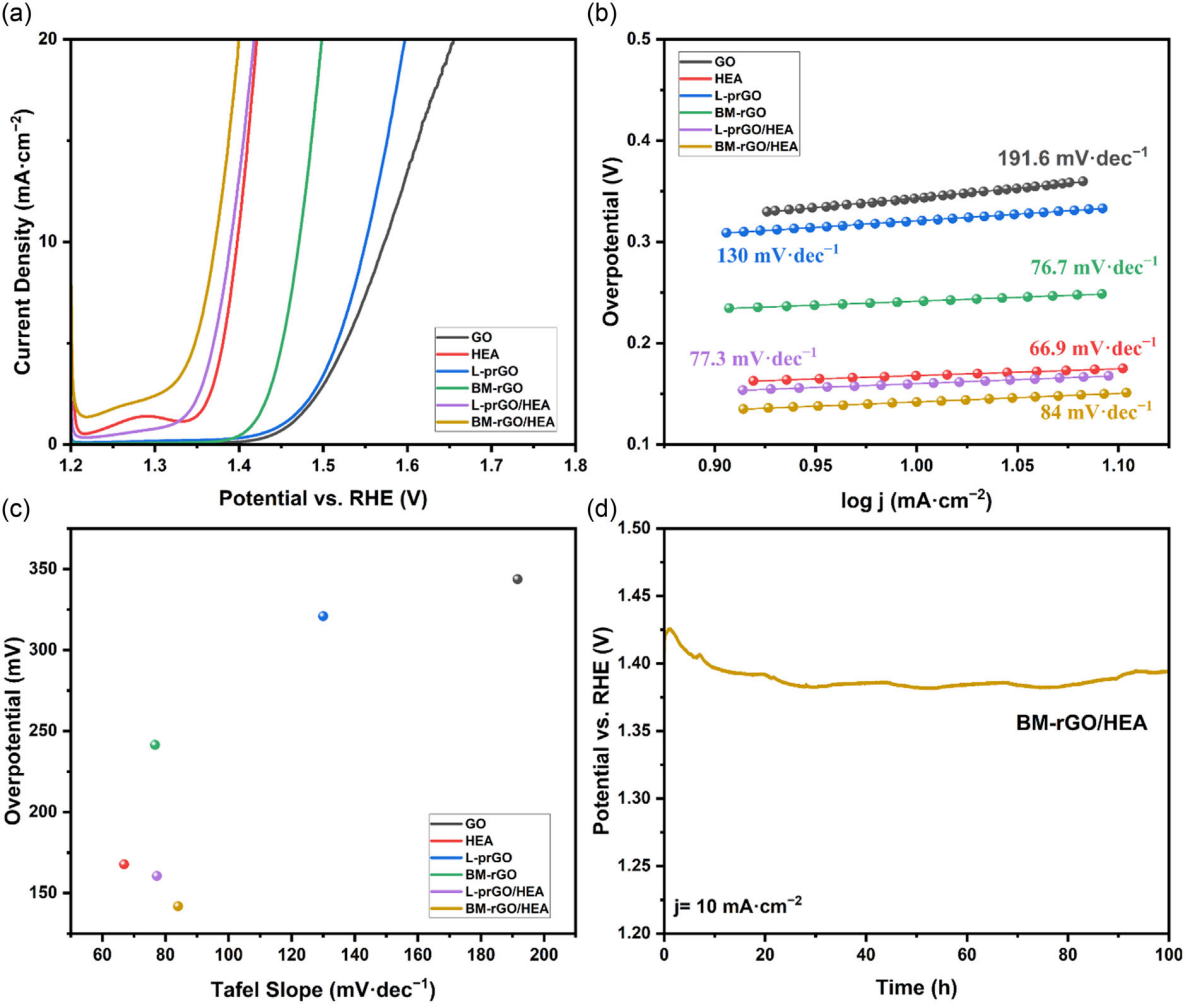
a) LSV curves corresponding to OER of different samples, b) the respective Tafel slopes for OER and c) Overpotential versus the Tafel slope. d) Chronopotentiometry curve corresponding to the long‐term (100 h) stability of BM‐rGO/HEA sample at a current density of 10 mA·cm^−2^.

The OER activity of the samples was evaluated in a solution of 1 M KOH as the electrolyte saturated with O_2_. As seen in **Figure** **4**a, LSV technique was employed to assess the OER activities of the samples. Figure 4b shows the corresponding Tafel plots for each sample, including the Tafel slopes, obtained from the LSV curve by the following equation.

n=a+blogj

where *n*, *j*, and *b* represent the overpotential, current density, and Tafel slope respectively. The overpotential required to reach a current density of 10 mA·cm^−2^ (η_10_) is plotted against the Tafel slope in Figure 4c. The values of η_10_ are displayed in **Table** **2** along with the corresponding Tafel slopes for each sample to gain further information about the OER kinetics. It is observed that the OER activity of GO is further improved after partial reduction via laser treatment and reduction through HEBM. This enhancement is mainly due to the increased conductivity, specific surface area and number of active sites in GO after reduction to rGO due to the removal of oxygen functional groups. Since more functional groups are removed in the HEBM process, more active sites are available on the rGO surface for the OER to take place, resulting in a lower overpotential compared to the partially reduced GO prepared by laser irradiation. As mentioned in the characterization section, trace amounts of Fe were detected in the BM‐rGO samples. This suggests that the improvement in the OER activity of the BM‐rGO sample compared to GO could be partially attributed to the presence of Fe atoms. However, since the HEA sample already contains larger amounts of Fe, the effect of ball‐mill‐induced Fe on the best performing electrocatalyst is insignificant. The NiCoFeMoW HEA powders have an inherently low overpotential due to the presence of highly active transition metals with several high valence states.^[^ ^45^ ^]^ The addition of HEA powders specifically changes the electronic state of surface atoms, resulting in enhanced adsorption of intermediate species.^[^ ^18^ ^]^ Finally, it is observed that simultaneous incorporation of HEA powders and processed GO results in the lowest overpotential of all, reaching a current density of 10 mA·cm^−2^ at 160.6 and 141.8 mV overpotentials for L‐prGO/HEA and BM‐rGO/HEA samples, respectively. Combining carbon‐based materials with transition metals generates a synergistic effect, significantly boosting the catalyst's activity.^[^ ^46^ ^]^ The enhanced OER activity of the rGO heterostructures can also be attributed to electron transfer from transition metals to rGO, facilitated by the favorable coordination of them with the graphene surface.^[^ ^47^ ^]^ According to the Tafel slopes presented in Table 2, the incorporation of HEA in rGO structure leads to improved OER kinetics. Furthermore, the long‐term stability measurements were carried out for the best sample (BM‐rGO/HEA) at a constant current density of 10 mA cm^−2^, where the electrode showed a stable overpotential for 100 h. These experiments reveal that the BM‐rGO/HEA heterostructure has led to a decrease both in overpotential and Tafel slope, while maintaining extraordinary stability.

**Table 2:** The overpotentials and Tafel slopes corresponding to each sample.

Sample	Overpotential at 10 mA·cm^−2^ [mV]	Tafel Slope [mV·dec^−1^]
GO	343.7	191.6
HEA	167.8	66.9
L‐prGO	320.9	130
BM‐rGO	241.5	76.7
L‐prGO/HEA	160.6	77.3
BM‐rGO/HEA	141.9	84

The ECSA of the samples was estimated using the double‐layer capacitance (C_dl_) approach. For this purpose, CV measurements were performed at five different scan rates ranging from 20 to 100 mV·s^−1^ within the potential window of 1.1–1.2 V versus RHE. The resulting CV curves are presented in Figure S1, Supporting Information. The ECSA was calculated using the following formula

$$ECSA=\frac{C_{dl}}{Cs}$$

**Figure 5:**
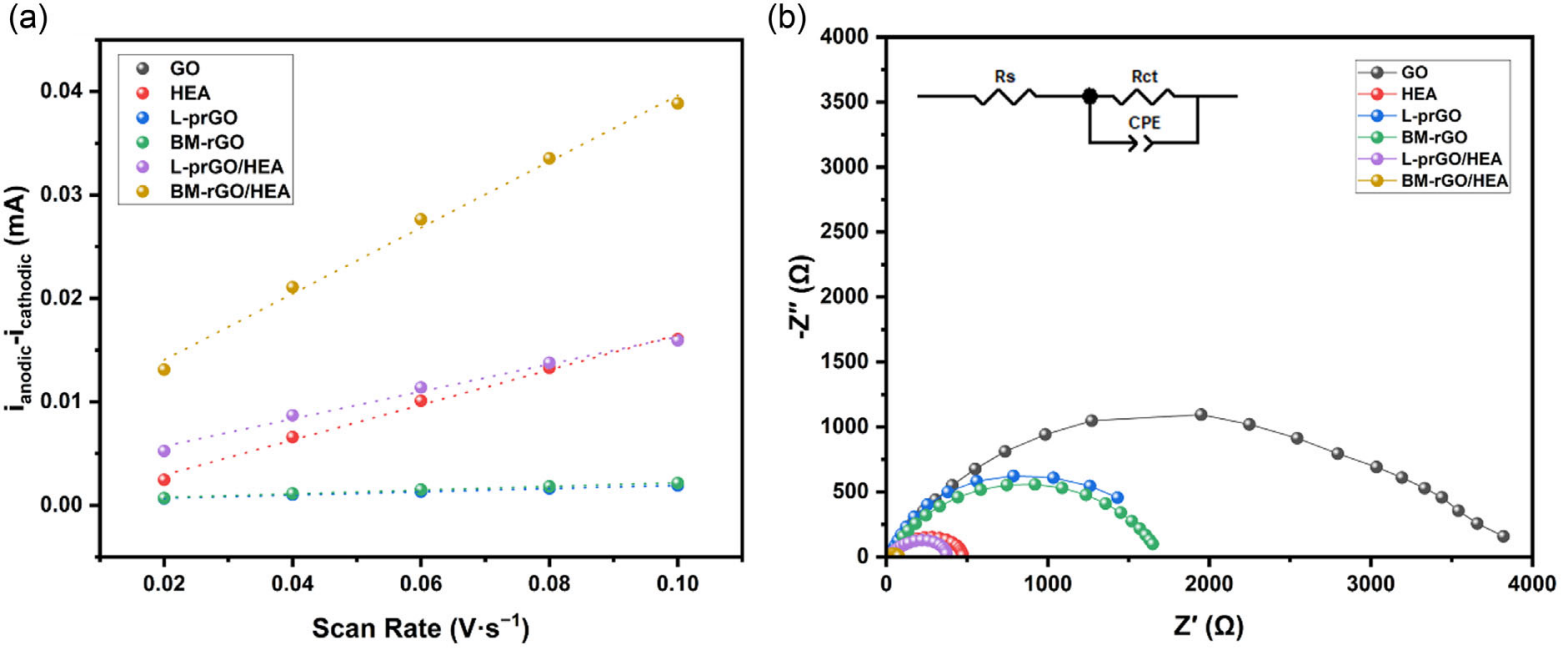
a) Non‐faradic current density versus scan rate curves for all samples, fitted linearly for ECSA calculations, b) EIS Nyquist plots corresponding to different samples. The inset shows the Randles equivalent circuit used for fitting the data.

In this equation, *C*_s_ denotes the specific capacitance of the material, while *C*_dl_ refers to the double‐layer capacitance obtained from the slope of the plot of charging current (*i*_*c*_) versus scan rate (*ν*). Based on prior studies, the *C*_*s*_ value in a 1 M KOH solution is reported to be 0.040 mF·cm^−^^2^.^[^ ^48^ ^]^ Based on the data obtained from Figure S1, Supporting Information, current density versus scan rate curves for all samples were plotted (**Figure** **5**a) and the corresponding *C*_*dl*_ and ECSA values for each sample are summarized in **Table** **3**.

**Table 3:** Double layer capacitance and corresponding ECSA values for the samples.

Sample Name	C_dl_ [mF]	ECSA [cm^2^]
GO	0.01	0.39
HEA	0.17	4.23
L‐prGO	0.01	0.39
BM‐rGO	0.02	0.45
L‐prGO/HEA	0.13	3.31
BM‐rGO/HEA	0.32	7.992

Among the samples, BM‐rGO /HEA exhibited the highest *C*_dl_ and ECSA values, suggesting a significant improvement in the electrochemically active surface area, which is expected to translate into superior catalytic activity. This superior ECSA can be ascribed to the synergistic effect between HEAs and rGO sheets prepared by HEBM.

EIS was utilized to investigate the interfacial and charge transfer characteristics of the electrodes. The resulting Nyquist plots for all samples are displayed in Figure 5b. The experimental data were fitted using the ZView software to extract key parameters, including solution resistance (*R*_*s*_), double‐layer pseudo‐capacitance (*C*_*dl*_), and most notably, charge transfer resistance (*R*_*ct*_), as detailed in **Table** **4**. In these plots, the intercept at high frequencies represents *R*_*s*_, while the diameter of the semicircle corresponds to *R*_*ct*_. The EIS data were modeled using a Randles equivalent circuit composed of *R*_*s*_, *R*_*ct*_, and a constant phase element (CPE) to simulate *C*_*dl*_.^[^ ^49^ ^]^ As shown in Table 4, the *R*_*ct*_ values decrease markedly from 3705 Ω for the pristine GO sample to 1687 Ω for L‐prGO and 1679 Ω for BM‐rGO. This improvement is linked to the elimination of oxygen‐containing functional groups on GO, which enhances the electrical conductivity of the electrode. The incorporation of HEAs further reduced the *R*_*ct*_ values due to their intrinsically high conductivity, yielding a value of 488 Ω. Notably, the hybrid materials L‐prGO/HEA and BM‐rGO/HEA exhibit even lower *R*_*ct*_ values of 415 Ω and 73.7 Ω, respectively. This pronounced decrease in charge transfer resistance following the integration of reduced GO and HEAs underscores the excellent electrocatalytic potential of these composite systems.

**Table 4:** EIS fitting data extracted from ZView software for all specimens.

Sample	R_s_ [Ω]	R_ct_ [Ω]	CPE‐T [μΩ^−1^s^n^]	CPE‐P
GO	9.6	3705	2.4 × 10^−5^	0.7
HEA	9.8	488	4.2 × 10^−5^	0.7
L‐prGO	10.7	1687	2.9 × 10^−4^	0.8
BM‐rGO	9.7	1679	1.8 × 10^−5^	0.7
L‐prGO/HEA	10.3	415	7.2 × 10^−5^	0.7
BM‐rGO/HEA	9.1	73.7	1.1 × 10^−4^	0.7

## 5 Conclusion

This study highlights the effectiveness of rGO‐supported NiCoFeMoW HEA powders in enhancing OER performance. Both laser irradiation and HEBM were employed for the reduction of GO, with HEBM demonstrating superior efficacy in lowering overpotentials and improving electrochemical activity. Structural characterization revealed that laser irradiation resulted in only partial reduction of GO, whereas HEBM achieved complete reduction, emphasizing its advantage over the laser‐based approach. The removal of oxygen‐containing functional groups during the reduction process induced two distinct structural modifications: “wrinkling,” attributed to localized thermal effects, which was more distinct in laser‐treated GO, and “folding,” caused by prolonged mechanical stress, which dominated in HEBM‐reduced GO. The latter suggests a more effective removal of oxygen groups and subsequent structural rearrangement. Furthermore, the partial recrystallization of the graphene lattice during the reduction of GO to rGO led to the formation of crystalline graphitic regions with short‐range ordering, as evidenced by discrete point‐like diffraction spots in HRTEM‐SAED patterns. Electrochemical analysis demonstrated that the OER activity of GO improved significantly following its partial reduction via laser treatment and further reduction through HEBM. This enhancement was attributed to increased conductivity, a larger specific surface area, and a greater number of active sites in rGO, resulting from the effective removal of oxygen functional groups. Since HEBM facilitated a higher degree of functional group removal compared to laser irradiation, it exposed more active sites on the rGO surface, thereby achieving a lower overpotential for OER compared to the partially reduced GO obtained through laser treatment. The incorporation of HEA powders further improved catalytic performance by modifying the electronic states of surface atoms, enhancing the adsorption of reaction intermediates. The combination of HEA with processed GO yielded the lowest overpotentials, reaching 10 mA·cm^−^^2^ at 160.6 mV for L‐prGO/HEA and 141.8 mV for BM‐rGO/HEA. This synergy significantly enhanced catalytic activity, driven by efficient electron transfer from transition metals to rGO. Overall, the rGO‐HEA heterostructures exhibited exceptional OER performance, achieving an overpotential of 141.8 mV at 10 mA·cm^−2^ along with enhanced charge transfer and long‐term stability.

## Conflict of Interest

The authors declare no conflict of interest.

## Supporting information

Supplementary Material

## Data Availability

The data that support the findings of this study are available from the corresponding author upon reasonable request.
