# Supplementary material for: Pulsed‐Laser and Mechanical Reduction of Graphene Oxide Combined with NiCoFeMoW High‐Entropy Alloys for Electrocatalytic Oxygen Evolution Reaction
Source: ChemSusChem. 2025 Jun 29;18(15):e202500466. doi: 10.1002/cssc.202500466 (PMC12302311; doi:10.1002/cssc.202500466)
Supplement: Supplementary file 1 — Supplementary Material [file CSSC-18-e202500466-s001.pdf]

Pulsed-laser and Mechanical Reduction of Graphene Oxide Combined with NiCoFeMoW High-Entropy Alloys for Electrocatalytic Oxygen Evolution Reaction

Hossein Mahdavi[a], *Omer Şamil Akcan*[b] ,*Yağız Morova*,[b, c], *M. Barış Yağcı* [c], *Uğur Ünal*[* c,d,e], *Hadi Jahangiri*[*c]

a-Materials Science and Engineering, Koç University, Sariyer, 34450, Istanbul, Turkey
b- Department of Physics, Istanbul Technical University, Istanbul, Turkey
c-Koç University Surface Science and Technology Center (KUYTAM), Koç University, Sariyer, 34450, Istanbul, Turkey
d-Department of Chemistry, Koç University, Sariyer, 34450, Istanbul, Turkey
e- Koç University Hydrogen Technologies Center (KUHyTech), Koç University, Sariyer, 34450, Istanbul, Turkey

Corresponding Author: Hadi Jahangiri
hjahangiri@ku.edu.tr
Ugur Unal
ugunal@ku.edu.tr

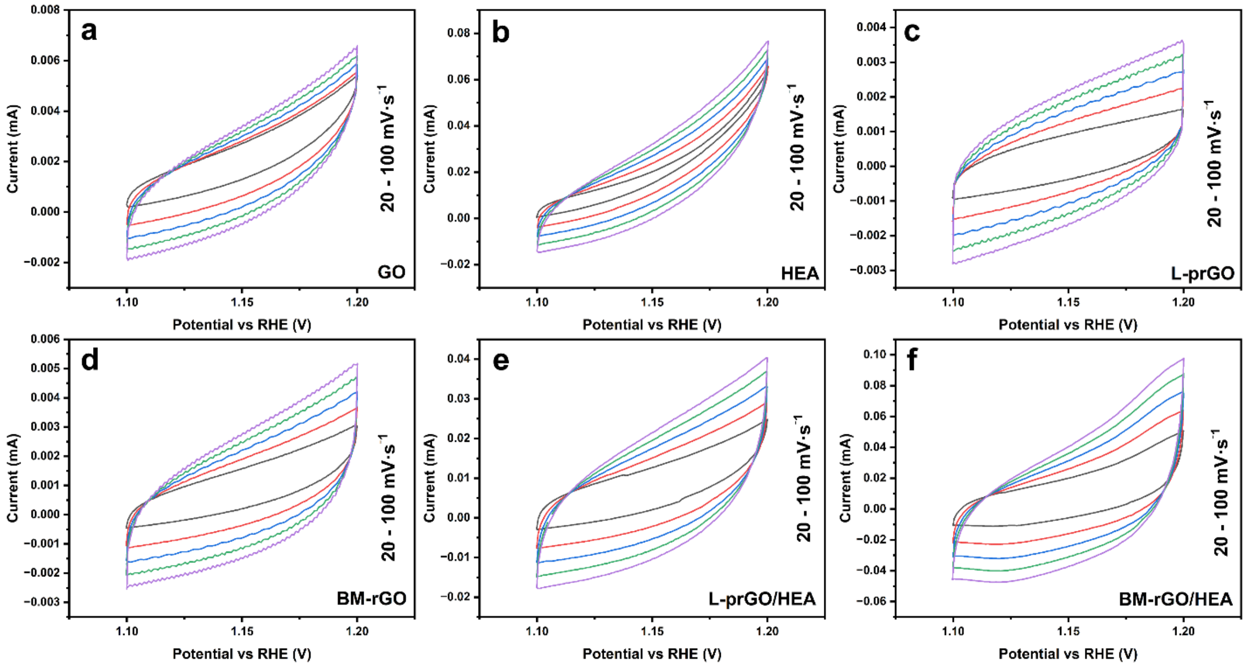

Figure S1. Cyclic voltammetry curves at scan rates ranging from 20 to 100 mV·s^{-1} for a) GO, b) HEA, c) L-prGO by laser, d) BM-rGO, e) L-prGO/HEA, and f) BM-rGO/HEA.

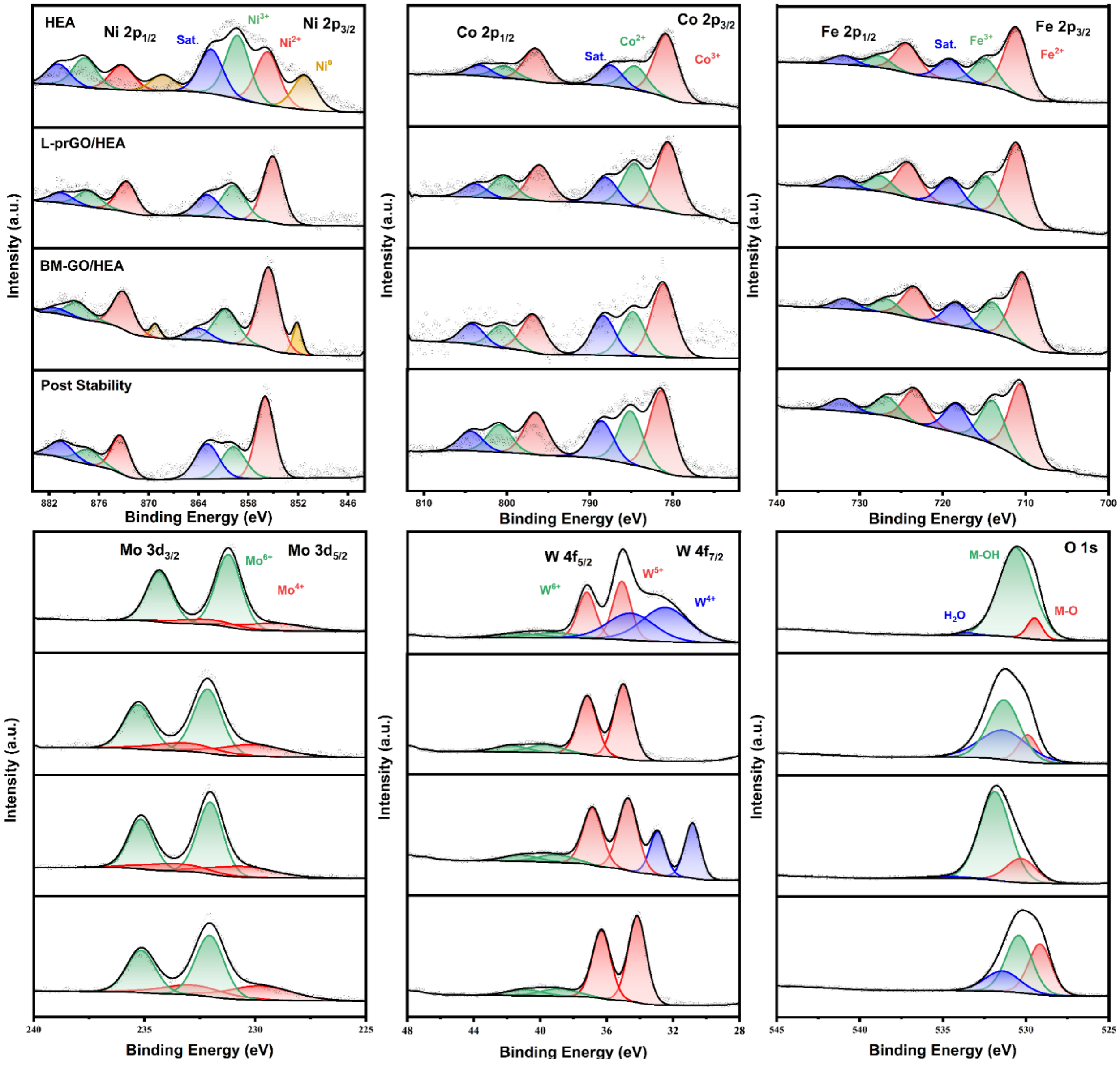

Figure S2 High-resolution X-ray photoelectron spectra of HEA-containing samples including HEA, L-prGO/HEA, BM-rGO/HEA and BM-rGO/HEA after long-term electrochemical stability measurements for OER.

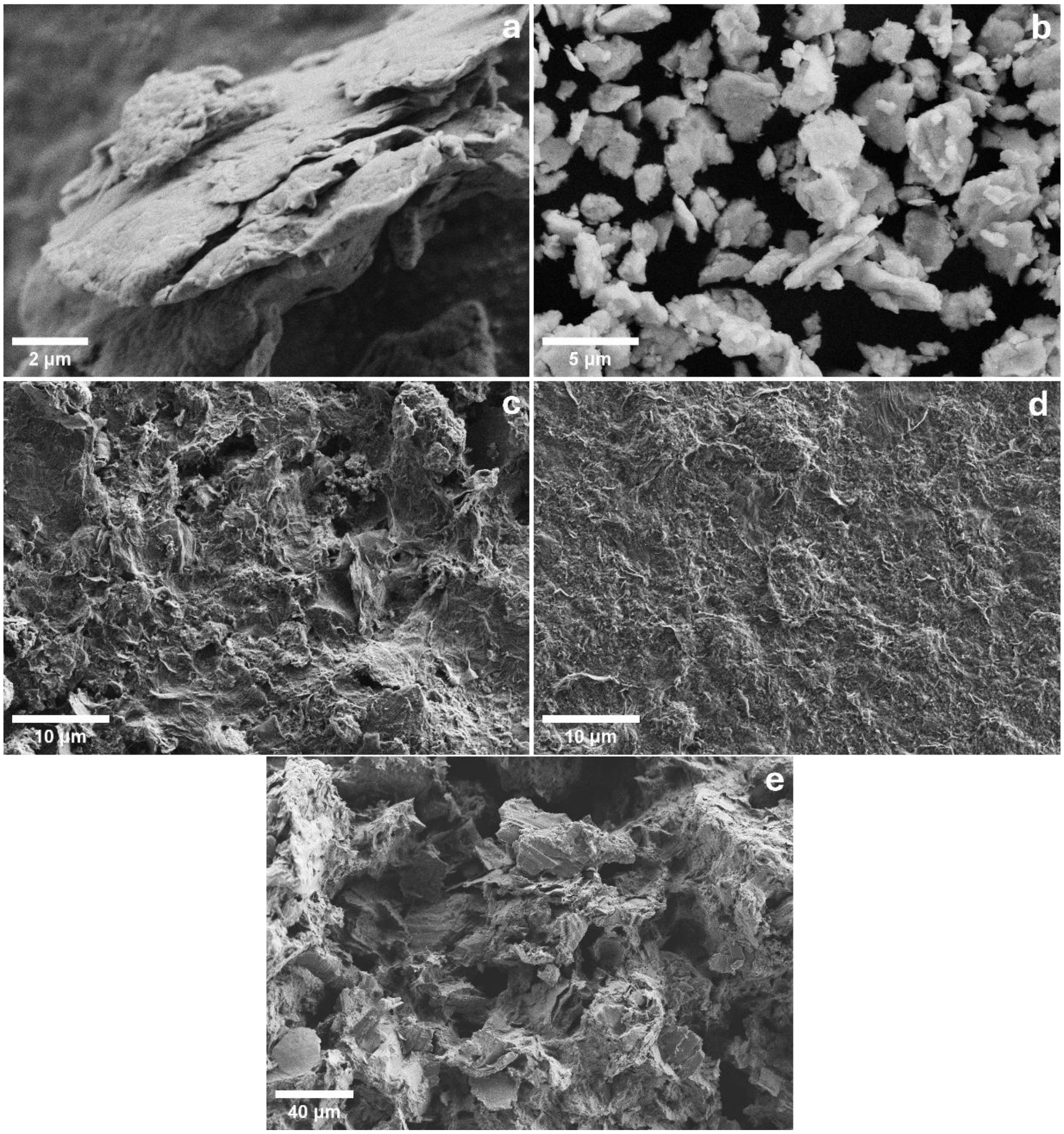

Figure S3 FE-SEM images of a) GO, b) HEA, c) L-prGO/HEA, d) BM-rGO/HEA, and e) BM-rGO/HEA after stability measurements.
